# Antibiotic prescribing in patients with self-reported sore throat

**DOI:** 10.1093/jac/dkw497

**Published:** 2016-12-19

**Authors:** Nishchay Mehta, Anne Schilder, Ellen Fragaszy, Hannah E. R. Evans, Oliver Dukes, Logan Manikam, Paul Little, Sarah C. Smith, Andrew Hayward

**Affiliations:** 1 Farr Institute of Health Informatics Research, UCL, 222 Euston Road, London NW1 2DA, UK; 2 evidENT, Ear Institute, UCL, 330 Gray's Inn Road, London WC1X 8DA, UK; 3 London School of Hygiene & Tropical Medicine, Keppel Street, London WC1E 7HT, UK; 4 Population, Policy and Practice, UCL Great Ormond Street, Institute of Child Health, London, UK; 5 Primary Care Group, Primary Care and Population Sciences Unit, University of Southampton, Aldermoor Close, Southampton SO16 5ST, UK; 6 London School of Hygiene & Tropical Medicine, 15-17 Tavistock Place, London WC1H 9SH, UK

## Abstract

**Objectives:** To investigate the predictors of general practitioner (GP) consultation and antibiotic use in those developing sore throat.

**Methods:** We conducted a prospective population-based cohort study on 4461 participants in two rounds (2010–11) from 1897 households.

**Results:** Participants reported 2193 sore throat illnesses, giving a community sore throat incidence of 1.57/ person-year. 13% of sore throat illnesses led to a GP consultation and 56% of these consultations led to antibiotic use. Participants most likely to have sore throats included women and children (e.g. school compared with retirement age); adjusted incidence rate ratio (aIRR) of 1.33 and 1.52, respectively. Participants with sore throat were more likely to consult their GP if they were preschool compared with retirement age [adjusted OR (aOR) 3.22], had more days of sore throat (aOR 1.11), reported more severe pain (aOR 4.24) or reported fever (aOR 3.82). Antibiotics were more often used by chronically ill individuals (aOR 1.78), those reporting severe pain (aOR 4.14), those reporting fever (aOR 2.58) or children with earache (aOR 1.85). Among those who consulted, males and adults who reported feeling anxious were more likely to use antibiotics; aOR 1.87 and 5.36, respectively.

**Conclusions:** Only 1 in 10 people who have a sore throat see a doctor and more than half of those attending get antibiotics. Further efforts to curb antibiotic use should focus on reducing initial GP consultations through public information promoting safe self-management, targeted at groups identified above as most likely to attend with sore throats.

## Introduction

More than one-third of all antibiotics prescribed for respiratory infections are because of sore throat,^1^ and one in two patients presenting to their general practitioner (GP) with these symptoms receive antibiotics.^2^^,^^3^ There is a national drive in the UK to reduce antibiotic prescribing,^4^ based on high-level evidence. Meta-analysis of randomized controlled trials of antibiotics for sore throat have shown that they only provide a small reduction in symptom severity and duration (1 day).^5^ UK observational studies have shown that a very large number of sore throats need to be treated with antibiotics to prevent a single complication of infection.^6–10^ Survey studies have shown that 1 in 5 patients taking broad-spectrum antibiotics and 1 in 12 taking narrow-spectrum antibiotics suffer side effects such as a rash or gastrointestinal upset,^11–15^ whilst observational studies using routine data have linked antibiotic use directly to drug resistance at international^16^^,^^17^and local^18^ levels.

So far, national guidance,^4^ GP education schemes^19–22^ and alternative prescribing strategies^23^ have focused on the GP consultation in an effort to reduce antibiotic use. However, the implementation of consultation-based strategies seems limited since antibiotic use and practice variation are not only high,^3^ but on the increase.^24^ There is therefore a continuing need to develop and target strategies that promote patient safe self-management of sore throat in the community and prevent unnecessary consultation. In fact, the last and only population-based study in England to investigate sore throat in the community was conducted in 1974 on 198 women aged 20–44 years over a 28 day observation period.^25–27^ This small and select sample, with short follow-up, undertaken four decades ago does not provide sufficient information on community burden and risk factors for sore throat, subsequent GP consultation and antibiotic use to inform new public information campaigns. We investigated these issues using a large population-based prospective national household survey including detailed reports of sore throat symptom profiles and GP consultation behaviour over two whole winter seasons in order to inform and target public health campaigns.

## Methods

### National household survey

One hundred and forty-six general practices volunteered to participate in the study through the primary care research network. GPs sent invitations to a random sample of their register. Around 10% of households invited to participate did so.^28^ In total, 4461 participants were recruited between the two waves of the survey during the winters of 2009/2010 and 2010/2011. Baseline questionnaires were used to collect basic demographic and medical history information. This included age, gender, postcode, ethnicity, pre-existing medical conditions and number of people living in the same household. Subsequently, participants were asked to complete prospective illness diaries for every day they experienced a ‘sore throat’. Data were collected daily through online surveys to reduce recall bias, with weekly telephone reminders to minimize missing data. Participants were asked about the presence and severity of sore throat and associated symptoms such as feeling feverish, headache, having muscle aches, cough, runny nose, blocked nose and sneezing. During illnesses participants completed a generic health-related quality-of-life measure (EQ5D-3L).^29^ Participants were also asked if they had sought help from their GP for their problems and whether they used antibiotics. The lead household responder was responsible for the scores entered for any persons in the house younger than 16, entering results by proxy for the very young and supervising more competent children with the process. Further details of the survey methodology have been described elsewhere.^30^

### Variable definition

A sore throat episode was defined as two or more consecutive days of moderate to severe sore throat self-reported by the participant.

An episode was assumed to have ended safely when the participant was free from symptoms for 2 days or more. A new episode was recorded after at least 7 days without symptoms. The relatively short 7 day window between episodes was allowed following sensitivity analyses between 14 and 21 day periods that showed no difference in overall sore throat incidence. If the participant consulted with the GP on multiple occasions during their illness only the first consultation was counted.

Age was categorized as preschool (0–4 years), early school (5–13 years), adolescence and young adult (14–24 years), early adulthood (25–44 years), middle age (45–65 years) and retirement age (>65 years). Ethnicity was defined as white British and other. Postcode was used to define participants’ geographical region in England (North, West Midlands, East Midlands and East of England, London, South-East and South-West), population density (defined as urban or rural) and index of multiple deprivation (categorized into national quintiles).

Sore throat and all associated symptoms (as described above) were reported absent, mild or moderate–severe. The EQ5D-3L consisted of five dimensions (mobility, self-care, usual activities, pain/discomfort and anxiety/depression), each with three levels of functioning (no problems, some problems and extreme problems). Both the index score and domain specific values were evaluated.

### Weighting

Since the survey was oversampled in South-West England and undersampled in those between 0 and 15 years we weighted analyses to age and regional structure of England to give locally and nationally representative estimates. The final weight also accounted for the method of sampling through households (i.e. participants from a larger household had a greater chance of being sampled compared with those from smaller households).

### Modelling

We used multilevel (patient and general practice level) Poisson regression models to evaluate sociodemographic determinants of sore throat, and logistic regression models to investigate sociodemographic and symptom profiles effects on GP consultation and antibiotic use.

Risk factors found to be significant in univariable analyses were modelled using multilevel multivariable models (at the level of the patient and general practice), in a forward stepwise fashion. We undertook tests for interaction between variables if both variables were independently related to the outcome and had a biologically plausible interdependent relationship to outcome. All statistical analyses were undertaken on Stata SE 13.1.

### Missing data

**Figure 1.**
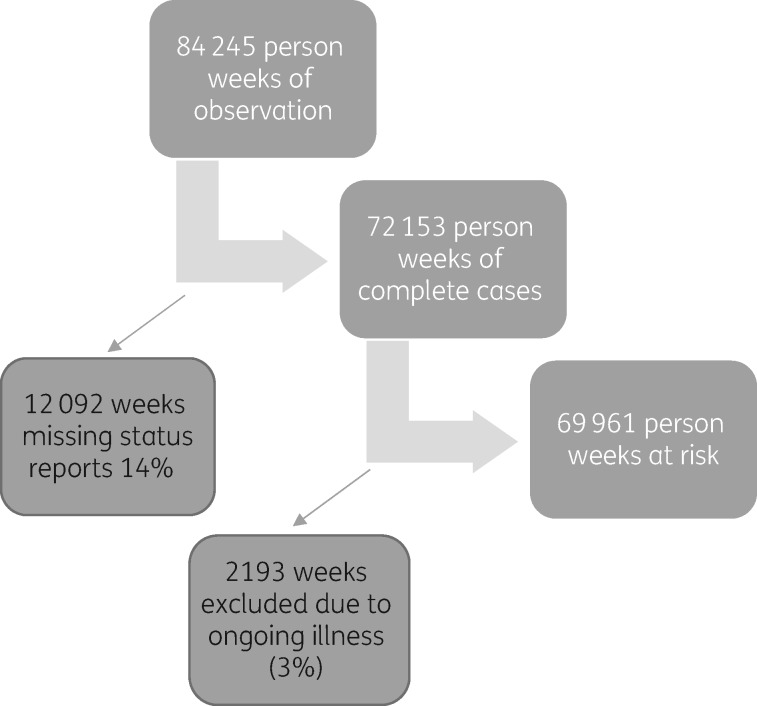
Loss to follow-up.

Status reports were missing for 12 092 weeks of the 84 245 weeks of person time, i.e. 14.4% of the data originally available for analysis were missing (see Figure 1). Sensitivity analyses were undertaken to explore the impact of these missing data. In one analysis we assumed that a week missing a status report was a week of no illness, whilst in another analysis we excluded the weeks with missing status reports from analysis (completed weeks analysis). There was minimal difference in the rates produced by either analysis and so we used completed weeks for subsequent analyses.

Loss to follow-up was defined by absence of weekly status reports in the last month of study. Less than 3% of the study population was lost to follow-up.

### Ethics

This study underwent ethical approval at Oxford A Research Ethics Committee (REC), 10/H0604/56. The study registration number was ISRCTN80214280.

## Results

### Main findings

The 4461 participants (median age 46 years, IQR 19–62, range 0–99) reported 2193 sore throat illnesses over 489 731 days at risk, giving a community sore throat incidence of 4.29 (95% CI 3.83–4.81)/1000 person-days and 1.57/person-year. In 280 of the 2193 (13%) sore throat illnesses, patients visited their GP, with the remainder self-managing their symptoms. Of those who consulted their GP, 157 (56%) used antibiotics.

### Sore throat incidence in the community

Key findings from univariable Poisson analyses (Table 1) include the increased risk of reporting a sore throat in females [incidence rate ratio (IRR) 1.32] and younger people (school age versus retirement age; IRR 1.70). White ethnicity, being a non-smoker, living in a large household (5 versus 2 people), receiving a flu vaccination that season and living in an urban region increased the risk of reporting a sore throat significantly.

**Table 1:** Sore throat incidence in the community and multilevel Poisson regression for sore throat risk factors

	Sore throat illnesses	Time at risk (days)	Univariable			Multivariable	
			IRR (95% CI)	incidence (episodes/1000 person days)	*P*	aIRR (95% CI)	*P*
0–4 years	87	22 240	1.41 (1.14–1.73)	3.96	<0.001	1.20 (0.92–1.56)	<0.001
5–13 years	283	56 819	1.70 (1.47–1.97)	4.78		1.52 (1.27–1.82)	
14–24 years	169	36 121	1.54 (1.30–1.82)	4.3		1.54 (1.25–1.89)	
25–44 years	533	92 143	1.88 (1.65–2.14)	5.28		1.79 (1.53–2.09)	
45–65 years	787	176 474	1.47 (1.30–1.65)	4.13		1.36 (1.18–1.57)	
>65 years	334	105 933	1	2.81		1	
Male	924	240 766	0.76 (0.71–0.83)	3.56	<0.001	0.75 (0.68–0.82)	<0.001
Female	1269	248 965	1	4.69		1	
North	214	48 046	0.96 (0.79–1.17)	3.83	0.07	–	–
West Midlands	116	27 076	0.93 (0.74–1.17)	3.71		–	
East and East Midlands	852	160 806	1.13 (0.98–1.30)	4.51		–	
London	155	29 088	1.18 (0.95–1.47)	4.70		–	
South-East	223	60 849	0.88 (0.72–1.09)	3.51		–	
South-West	633	163 866	1	3.99		–	
Smokers	88	28 809	0.60 (0.20–0.73)	2.61	<0.001	–	–
Non-smokers	1737	366 203	1	4.35		–	–
White ethnicity	2028	422 601	1	4.36	0.04	–	–
Non-white ethnicity	76	15 242	0.79 (0.63–0.99)	3.45		–	
One person in household	116	27 988	1.03 (0.86–1.24)	3.82	<0.001	–	–
Two people in household	898	221 787	1	3.71		–	
Three people in household	337	73 342	1.15 (1.02–1.30)	4.70		–	
Four people in household	596	117 010	1.30 (1.18–1.44)	5.32		–	
Five people in household	201	41 532	1.23 (1.06–1.42)	5.03		–	
Six people in household	45	8073	1.00 (0.75–1.32)	4.09		–	
Received flu vaccination this year	543	110 449	1.02 (0.93–1.11)	4.17	<0.001	–	–
Did not receive flu vaccine this year	1608	368 198	1	4.09		–	
IMD 1 (most deprived)	59	12 889	0.99 (0.78–1.25)	4.38	0.11	–	–
IMD 5 (least deprived)	730	144 627	1	4.43		–	
Urban	904	183 773	1.06 (0.96–1.16)	4.45	<0.001	–	–
Rural	1275	270 848	1	4.20		–	
Well	1836	374 381	1	4.30	0.6	–	–
Chronic illness	328	71 249	0.96 (0.84–1.10)	4.13		–	–

IMD, index of multiple deprivation (IMD 5 = least deprived quintile).

In the multivariable Poisson analysis only female gender [adjusted IRR (aIRR) 1.33] and being young (aIRR 1.52) increased the risk of reporting a sore throat illness (Table 1). Social deprivation, ethnicity, chronic illness, population density, influenza vaccination and smoking were not related to risks of reporting a sore throat. Interaction testing showed a significant interaction between gender and age with females between the ages of 5 and 44 more likely to report sore throats.

### GP consultation behaviour for those with sore throat illnesses

Results from univariable logistic analyses are summarized in Table 2. Sociodemographic factors increasing the risk of a person with sore throat consulting their GP included being young. Disease factors increasing the risk of GP consultation for sore throat included duration of sore throat episode, severity of pain, ear ache, fever and cough. A reduction in health-related quality of life, as measured by increases in certain EQ5D-3L subdomain scores (usual activities, self-care, mobility and anxiety) was associated with increased chance of GP consultation. Multivariable logistic analysis showed that being young [adjusted OR (aOR) 3.22], days of sore throat (aOR 1.11), extreme pain (aOR 4.24) and fever (aOR 3.82) significantly increased the risk of participants consulting their GP for a sore throat. Tests of interaction between age and sore throat symptoms (duration, pain and fever) were not significant.

**Table 2:** Risk of GP consultation for sore throat and multilevel logistic regression of risk factors for GP consultation

Variable	GP visits	Total sore throat episodes	%	Univariable analysis		Multivariable analysis	
				OR (95% CI)	*P*	aOR (95% CI)	*P*
Chronically ill	65	442	14.71	1.81 (1.26–2.60)	0.001	—	—
Well	211	2425	8.70	1		—	
0–4 years	37	87	42.53	3.38 (1.92–5.96)	<0.001	3.22 (1.81–5.73)	<0.001
5–13 years	37	283	13.07	1.26 (0.71–2.23)		1.25 (0.68–2.27)	
14–24 years	27	169	15.98	1.58 (0.82–3.04)		1.99 (0.90–4.40)	
25–44 years	51	533	9.57	0.93 (0.56–1.53)		0.99 (0.60–1.65)	
45–65 years	90	787	11.44	1.04 (0.65–1.66)		1.04 (0.60–1.77)	
>65 years	38	334	11.38	1		1	
Female	169	1269	13.32	1	0.3	1	0.24
Male	111	924	12.01	0.85 (0.63–1.16)		0.82 (0.59–1.14)	
Rural	180	1275	14.12	1	0.1	—	—
Urban	100	904	11.06	0.77 (0.57–1.05)		—	
Non-white	16	76	21.05	1.76 (0.95–3.25)	0.07	—	—
White	252	2028	12.43	1		—	
IMD 1 (most deprived)	6	59	10.17	0.71 (0.31–1.61)	0.63	—	—
IMD 5 (least deprived)	106	730	14.52	1		—	
Smokers	10	88	11.36	1.04 (0.48–2.28)	0.91	—	—
Non-smokers	194	1737	11.17	1		—	
Duration (days)	271	2193	12.36	1.10 (1.08–1.13)	<0.001	1.11 (1.08–1.14)	<0.001
Earache	101	424	23.82	2.99 (2.19–4.09)	<0.001	—	—
No earache	179	1684	10.63	1		—	
Any cough	236	1612	14.64	2.24 (1.49–3.36)	<0.001	—	
No cough	44	581	7.57	1		—	
Fever	115	475	24.21	4.87 (3.34–7.09)	<0.001	3.82 (2.64–5.56)	<0.001
No fever	94	1522	6.19	1		1	
EQ5D index score	271	2193	12.36	0.10 (0.05–0.19)	<0.001	—	—
Extreme pain	24	61	39.34	6.95 (3.23–14.97)	<0.001	4.24 (1.81–9.94)	0.004
Some pain	47	388	12.11	1.48 (0.94–2.33)		1.38 (0.83–2.29)	
No pain	209	2449	8.53	1		1	
Extreme loss of usual activity	28	84	33.33	5.44 (2.92–10.12)	<0.001	—	—
Some loss of usual activity	34	222	15.32	1.97 (1.13–3.41)			
No loss of usual activity	218	2592	8.41	1		—	
Extreme inability to self-care	9	16	56.25	12.77 (3.32–49.08)	<0.001	—	—
Some inability to self-care	11	40	27.50	3.77 (1.41–10.10)			
Normal self-care	260	2842	9.15	1		—	
Extreme loss of mobility	20	50	40.00	6.95 (4.09–11.80)	<0.001	—	—
Some loss of mobility	18	83	21.69	2.89 (1.39–6.01)			
Normal mobility	242	2765	8.75	1		—	
Severe anxiety	4	11	36.36	5.67 (1.24–25.98)	0.001	—	—
Some anxiety	22	111	19.82	2.45 (1.32–4.55)			
No anxiety	254	2776	9.15	1		—	

IMD, index of multiple deprivation (IMD 5 = least deprived quintile).

### Antibiotic prescribing for sore throat

Initial multivariable multilevel logistic analyses of antibiotic use in relation to all sore throat episodes showed that chronic illness was the only patient factor related to antibiotic use (aOR 1.78). Sore throat symptom features related to antibiotic use included earache (aOR 1.85), fever (aOR 2.58) and extreme pain (aOR 4.14) (see Table 3 for all results). Further testing only showed an interaction between age and earache: children were more likely to receive antibiotics if they reported a sore throat as well as earache compared with adults.

**Table 3:** Risk of antibiotic use amongst those with sore throat and multilevel logistic regression risk factors for antibiotic use

Variable	Antibiotics taken	Total sore throat episodes	%	Univariable analysis		Multivariable analysis	
				OR (95% CI)	*P*	aOR (95% CI)	*P*
Chronically ill	56	328	17.07	1.99 (1.37–2.88)	<0.001	1.78 (1.24–2.62)	0.004
Well	167	1836	9.10	1		1	
0–4 years	23	87	26.44	2.61 (1.21–5.63)	0.1	2.23 (0.99–5.01)	0.23
5–13 years	25	283	8.83	1.08 (0.54–2.14)		1.01 (0.47–2.19)	
14–24 years	19	169	11.24	1.36 (0.62–2.97)		1.61 (0.74–1.49)	
25–45 years	46	533	8.63	1.08 (0.60–1.95)		1.08 (0.58–2.03)	
46–65 years	84	787	10.67	1.27 (0.75–2.17)		1.22 (0.70–2.11)	
>65 years	29	334	8.68	1		1	
Female	132	1269	10.40	1	0.94	1	0.82
Male	94	924	10.17	0.99 (0.70–1.39)		1.04 (0.73–1.49)	
Rural	137	1275	10.75	1	0.60	—	
Urban	89	904	9.85	0.91 (0.63–1.31)		—	
Non-white	14	76	18.42	1.75 (0.81–3.78)	0.15	—	
White	206	2028	10.16	1		—	
IMD 1 (most deprived)	4	59	6.78	0.69 (0.24–1.98)	0.85	—	
IMD 5 (least deprived)	73	730	10.00	1		—	
Smokers	11	88	12.50	1.21 (0.54–2.74)	0.64	—	
Non-smokers	215	2105	10.21	1		—	
Duration (days)	2193	2193	100.00	1.09 (1.06–1.11)	<0.001	1.08 (1.06–1.10)	
Earache	80	424	18.87	2.36 (1.68–3.29)	<0.001	1.85 (1.25–2.75)	0.002
No earache	146	1684	8.67	1		1	
Any cough	191	1612	11.85	1.90 (1.18–3.03)	0.008	—	
No cough	35	572	6.12	1		—	
Fever	83	367	22.62	3.02 (2.19–4.17)	<0.001	2.58 (1.74–3.82)	<0.001
No fever	143	1826	7.83	1		1	
EQ5D index score	2193	2193	100.00	0.13 (0.06–0.28)	<0.001	—	
Extreme pain	21	52	40.38	5.88 (2.81–12.30)	<0.001	4.14 (1.74–3.81)	0.002
Some pain	37	331	11.18	1.33 (0.82–2.15)		1.18 (0.74–1.89)	
No pain	168	1810	9.28	1		1	
Extreme loss of usual activity	20	76	26.32	3.34 (1.77–6.30)	<0.001	—	
Some loss of usual activity	27	191	14.14	1.62 (0.94–2.78)		—	
No loss of usual activity	179	1926	9.29	1		—	
Extreme inability to self-care	9	12	75.00	13.93 (3.20–60.66)	<0.0004	—	
Some inability to self-care	10	32	31.25	3.44 (1.18–10.02)		—	
Normal self-care	207	2149	9.63	1		—	
Extreme loss of mobility	16	45	35.56	5.20 (2.89–9.38)	<0.001	—	
Some loss of mobility	12	73	16.44	1.92 (0.93–3.96)		—	
Normal mobility	198	2075	9.54	1		—	
Severe anxiety	4	9	44.44	6.15 (1.31–28.96)	0.004	—	
Some anxiety	18	100	18.00	2.48 (1.26–4.88)		—	
No anxiety	204	2084	9.79	1		—	

IMD, index of multiple deprivation (IMD 5 = least deprived quintile).

Subsequent analyses of antibiotic use were specifically restricted to the sore throat episodes which resulted in GP consultation. Results from multivariable multilevel logistic analyses are summarized in Table 4 and showed that being male (aOR 1.87) and self-reporting greater anxiety (aOR 5.36) significantly increased the risk of receiving antibiotics following a visit to the GP for a sore throat. Interaction testing between age and anxiety showed that adults who reported anxiety were more likely to receive antibiotics compared with children.

**Table 4:** Risk of antibiotic use amongst those who consult their GP for sore throat and multilevel multivariable logistic regression for risk factors for antibiotic use

Variable	Antibiotic taken	GP consultations	%	Multivariable analysis	
				aOR (95% CI)	*P*
0–4 years	16	37	43.243	0.50 (0.16–1.53)	0.51
5–13 years	18	37	48.649	0.65 (0.21–2.07)	
14–24 years	16	27	59.259	1.09 (0.32–3.70)	
25–44 years	31	51	60.784	1.22 (0.39–3.81)	
45–65 years	54	90	60	0.98 (0.42–2.30)	
>65 years	22	38	57.895	1	
Female	88	169	52.071	1	0.05
Male	69	111	62.162	1.87 (1.00–3.48)	
Anxiety	20	26	76.923	5.36 (1.44–19.91)	0.01
No anxiety	137	254	53.937	1	

## Discussion

This study demonstrates a high incidence of sore throat in the community (1.57 episodes/person-year) with the majority of sore throat illnesses (87.2%) safely managed without GP consultation or prescription. Younger age not only increased the risk of reporting a sore throat (school compared with retirement age: aIRR 1.52) but also increased the chance of consulting a GP once a sore throat was reported (school age compared with retirement age: aOR 4.05). Whilst women were more likely to report a sore throat (aIRR 1.33) they were just as likely to consult their GP as men once a sore throat had been reported. Participants were more likely to consult their GP for a sore throat if their illness was associated with more days of sore throat (aOR 1.11), extreme pain (aOR 4.24) or fever (aOR 3.82). Despite some targeting of antibiotic use in sore throat illnesses to those with chronic illness (aOR 1.78), extreme pain (aOR 4.14) and fever (aOR 2.58) more than half (57%) of those attending still received antibiotics.

This is the largest population-based survey of sore throat to date, weighted to represent the national population. The prospective nature of data collection, through daily health diaries and weekly telephone calls, allowed us to reduce the recall bias inherent in retrospective interview studies. This survey method also allowed us to reduce our missing data (14% missing weekly status reports). Sensitivity analyses of different ways of accounting for our missing data showed no change in our conclusions. In contrast to electronic healthcare record studies, we were able to accurately assess the role of associated symptoms and health-related quality of life in the management of sore throat episodes, by using detailed disease profile questions. In addition, we were able to directly measure antibiotic use through patient self-report, rather than indirectly through prescription rates. This study has three limitations. Firstly, very young children, residents of North England and those of lowest socioeconomic status were under-represented in the study population. Therefore, the survey was weighted to allow the incidence to be more representative of local and national populations. Secondly, 1 year of the study was conducted in a pandemic influenza outbreak year when there was considerable media coverage, which may have increased symptom vigilance and affected consultation behaviour. Lastly, we did not have data on GP prescription rates. Therefore, the rate of delayed antibiotic prescribing cannot be ascertained. We have only reported whether antibiotics were taken or not. We have therefore not been able to evaluate the effectiveness of the delayed antibiotic prescribing strategy. Even if a delayed prescribing strategy was used frequently, the fact that more than half of sore throat consultations ended in antibiotic use shows further strategies are needed.

The only other prospective population-based study in England was undertaken in Lambeth in 1974 on 198 women, aged 20–44, who were asked to keep a prospective health diary for 28 days each. During the observation period 90 sore throat episodes were reported [an annual sore throat incidence of 5.9 (95% CI 4.7–7.3) sore throat episodes per person-year] with 33 subsequent GP consultations [a consultation rate of 37% (95% CI 25%–51%)]. However, since this was a small study in a select population it is difficult to draw meaningful comparisons. Our results add to the growing body of evidence from general population studies of respiratory infections^28^^,^^31^ that the majority of sore throats are managed safely in the community. The rate of antibiotic prescribing for sore throat found in our study is comparable to previous primary care studies.^2^^,^^3^ However, these studies retrospectively defined their sore throat population using GP diagnostic codes which are known to be inconsistently used for this condition^32^ and indirectly approximated antibiotic usage through prescription rates. The present study was able to prospectively define its population based on symptoms experienced and to directly measure antibiotic use through patient self-report.

We found that GP consultation for sore throat was predicted by young age. Studies of all respiratory infections also confirm that young age is a major driver for primary care use in the UK^33^ with qualitative studies showing that parents’ decision to bring their children to the GP is influenced by perceived threat, disease severity, the perceived benefits of consulting, and an expectation of assessment, information, advice or treatment.^33–37^ Our study also found that severity of throat pain, duration of sore throat and presence of fever were related to GP consultation. Explanations for these results can be offered from qualitative research into people with acute sore throat^38^ and respiratory tract infections^31^ showing that most commonly seek help from their GP for pain relief, perceived symptom severity and non-resolution of symptoms. Amongst those who see their GP for sore throat, being male and self-reporting anxiety were the only factors related to an increased chance of taking antibiotics. However, it should be noted that whilst women were more likely to report a sore throat, gender had no overall relationship between sore throat and antibiotic use (see Table 3). Having controlled for pain and duration, increased anxiety was still an important predictor for receiving antibiotics in adults. Whilst patient anxiety has not previously been studied as a driver for high antibiotic prescribing, GPs have been shown to prescribe antibiotics under perceived patient pressure.^39^^,^^40^ Further qualitative research could be undertaken to explore the mechanism through which patient anxiety leads GPs to prescribe antibiotics for sore throat more frequently. Our study shows no relationship between sore throat severity, fever, cough, severity of pain, reduced health-related quality of life on antibiotic use for those who consulted. Whilst these factors affected participants’ decisions to consult, they did not appear to affect subsequent antibiotic prescription in this cohort. Qualitative studies investigating high antibiotic use have focused on GP behaviour and have shown a complex interplay of factors including perceived clinical need,^40^^,^^41^ perceived patient/parent pressure for antibiotics,^39^ clinical uncertainty^42–44^ and the desire to maintain a good relationship with the patient (or parent).^42^^,^^43^^,^^45^

Interventions to reduce antibiotic prescribing such as GP outreach programmes,^46^ and more recently web-based GP education programmes,^47^ have shown some benefit but have not been widely implemented in the UK. In fact, antibiotic prescribing in primary care increased by 6.2% from 2011 to 2014,^24^ and even though there was a 5.4% reduction from 2015 to 2016,^48^ there were more antibiotic prescriptions in 2016 compared with 2011. In addition, review of the literature shows that a multifaceted approach that includes patients and the public has the greatest impact on antibiotic prescription.^49^^,^^50^ Therefore, there is an urgent need to design additional strategies targeted at risk groups within the general population to help them better understand and self-manage sore throat, minimizing unnecessary GP consultations and subsequent antibiotic prescribing. Whilst the general poster and leaflet campaign in England encouraging the public not to take antibiotics for common colds had little effect on antibiotic prescriptions,^51^ studies of targeted patient education have shown reductions in antibiotic use.^52–54^ Public information particularly targeted at groups that our study found to be at risk of sore throat or most likely to consult, such as women and children, could aim to promote safe self-management and reduce anxiety associated with sore throat. This could emphasize that moderate to severe pain, prolonged duration and fever are frequent self-limiting features of sore throat. There is also a need to moderate the messages by highlighting ‘red-flag’ symptoms (e.g. trismus, difficulty breathing, neck swelling, torticollis), so the threshold for help-seeking behaviour may align more accurately with patients who would benefit from antibiotics in sore throat infections.^55–57^ Messages which highlight the fact that antibiotics provide minimal additional benefit over and above simple over-the-counter medications and often result in unwanted side effects may be more effective than those focusing on the less immediate problem of resistance.^58^ Tackling the problem of over-prescribing of antibiotics in primary care requires development of joined-up strategies targeting the general public, those who present with sore throat and their doctors.

## Funding

This project was funded by the Medical Research Council and the Wellcome Trust.

Funding bodies had no role in: study design; collection, analysis and interpretation of data; writing of the report; and decision to submit the article for publication. Researchers were independent from funders.
